# Outcome and risk factors of complications after cranioplasty with polyetheretherketone and titanium mesh: A single-center retrospective study

**DOI:** 10.3389/fneur.2022.926436

**Published:** 2022-09-21

**Authors:** Shun Yao, Qiyu Zhang, Yiying Mai, Hongyi Yang, Yilin Li, Minglin Zhang, Run Zhang

**Affiliations:** ^1^Department of Neurosurgery, Zhujiang Hospital, The Second School of Clinical Medicine, Southern Medical University, The National Key Clinical Specialty, The Engineering Technology Research Center of Education Ministry of China, Guangdong Provincial Key Laboratory on Brain Function Repair and Regeneration, The Neurosurgery Institute of Guangdong Province, Guangzhou, China; ^2^Department of Neurosurgery, Huashan Hospital, Fudan University, Shanghai, China; ^3^The Second School of Clinical Medicine, Southern Medical University, Guangzhou, China

**Keywords:** titanium mesh, polyetheretherketone, complications, risk factors, retrospective studies

## Abstract

**Background:**

To compare the incidence of complications and constructive effects of cranioplasty with polyetheretherketone (PEEK) and titanium mesh after decompressive craniectomy, and to further explore potential risk factors of postoperative and post-discharge complications.

**Methods:**

A retrospective study was conducted on 211 patients who underwent PEEK or titanium mesh cranioplasty in the Department of Neurosurgery of Zhujiang Hospital, Southern Medical University, between July 2017 and September 2021. Demographic data, imaging data, and postoperative complications were recorded and statistically analyzed. Long-term effects and satisfaction degree were evaluated based on following-up telephone survey. Univariate and multivariate logistic regression models were used to analyze risk factors of postoperative and post-discharge complications of PEEK and titanium cranioplasty.

**Results:**

The total postoperative complication rates of the PEEK and titanium mesh groups were 38.7 and 51.4% (*p* = 0.063), and post-discharge complication rates were 34.7 and 36.0% (*p* = 0.703), respectively. The incidence of pneumocephalus during hospitalization (33.3% vs. 6.6%, *p* < 0.001) and epidural effusion in the titanium mesh group were significantly higher than that in the PEEK group (18.0 vs. 6.6%, *p* = 0.011). Patients in PEEK group were less likely to occur subcutaneous effusion after discharge than in TI group (2.0 vs. 10.5%, *p* = 0.013). Multivariate logistic regression analysis revealed a history of ventriculoperitoneal shunt (VPS) before CP was an independent risk factor for postoperative overall complications (*p* = 0.023). Either superficial (*p* < 0.001) or intracranial infection (*p* = 0.001) was a risk factor for implant failure. Depressed skull defects (*p* = 0.024) and cranioplasty with titanium cranioplasty (*p* < 0.001) were associated with increased incidence of early pneumocephalus.

**Conclusion:**

There were no differences in overall postoperative and post-discharge complication rates between the titanium mesh and PEEK. A history of VPS before cranioplasty was an independent risk factor for postoperative overall complications, and infection was a risk factor for implant failure. Finally, depression skull defects and titanium mesh implants increased the incidence of postoperative pneumocephalus. Our results aim to promote a better understanding of PEEK and titanium cranioplasty and to help both clinicians and patients make better choices on implant materials.

## Introduction

Cranioplasty (CP) is a delayed neurosurgical procedure for skull defect reconstruction in patients who have undergone decompressive craniectomy (DC) to treat intracranial hypertension caused by trauma, intracranial hemorrhage, or neoplasms (1). CP has been a routine surgery in neurosurgery departments for decades, which can not only provide protective and cosmetic benefits, but also achieve significant neurological and cognitive improvement (2, 3). However, CP still faces many challenges, including the choice of appropriate implant materials, optimal timing, and the reduction of postoperative and post-discharge complications, on which surgeons should focus to avoid reoperation (4–7). Discharged patients should be followed up to assess long-term constructive effects, especially the shape changes of the repair site.

Several implant materials for CP have been investigated, including autologous bone, titanium mesh, polyetheretherketone (PEEK), and polymethyl methacrylate (PMMA) (8–12). Considering the limitations of autologous bone grafts, including the shortage of graft sources and unpredictable bone resorption, autologous bone graft was found to be unsatisfactory for some patients' recovery, particularly those with large cranial defects. In addition, reoperation is sometimes required to treat complications (12, 13). Compared with alloplastic grafts, autologous bone grafts have a higher rate of reoperation; however, the infection rates show no difference between the two materials (12), and PEEK appears to have the lowest risk of reoperation (14).

Accordingly, alloplastic materials such as titanium mesh, PEEK, and PMMA may be better. Titanium mesh is considered to have excellent biocompatibility, low cost, and satisfactory cosmetic effect, especially with three-dimensional (3D) custom-made mesh (15). However, it has insufficient protection against traumatic force and has potential exposure risk (6, 16), and as a thermoconductor, it may cause scalp paresthesia, which may also lead to scattering artifacts on conventional imaging, and this depends on mixed metal concentration (12, 14). PEEK is a hard synthetic polymer with good histocompatibility, stable chemical properties, stable temperature after heating, and elastic modulus close to the cortical bone. Therefore, PEEK may be a good option for the treatment of large cranial defects, which can achieve perfect symmetry and good functional results (7, 16–19). Nevertheless, PEEK lacks integration with the surrounding bone and still has complications, while having the highest cost among all alloplastic materials (9, 20, 21). Jack stated that PEEK has a lower risk of reoperation than titanium mesh (14). However, Jeremie's findings indicated that PEEK has a significantly higher local complication rate and the highest ultimate graft failure rate compared with all other implant types including titanium mesh and PMMA (20). A recent multicentral, randomized controlled study that assesses the long-term outcome of PEEK and titanium mesh CP has just initiated (22). However, studies comparing the outcomes of different CP materials are still limited, which is warranted in the next few years for the global neurosurgery departments.

This retrospective study aims to compare the complication rate, and the long-term constructive effect of PEEK and titanium mesh, and to further explore potential predictors of postoperative and post-discharge complications. Compared with similar research, we investigated the complications after CP, including common and less studied complications, and we explored relationship with overall clinical variates of interest. Finally, we provide some advice for the future clinical practice of CP.

## Materials and methods

### Clinical data selection and extraction

This retrospective study was approved by the Zhujiang Hospital Institutional Review Board. Patient selection was finished in January 2022; at that time, two authors repeatedly checked the medical records of 211 patients who underwent CP in our department from July 2017 to September 2021 and grouped them according to CP material (PEEK or titanium mesh). The inclusion criteria were as follows: (a) an exact history of DC due to traumatic brain injury, subdural hematoma, hemorrhagic or ischemic stroke, epidural hematoma, and subarachnoid hemorrhage, diagnosed by computed tomography (CT); (b) exact indications of CP without surgical contraindications; (c) CT examination 24–48 h after CP; d) patients followed up more than 6 months after cranioplasty. Exclusion criteria were as follows: (a) CT examination not performed 24–48 h after operation; (b) suspected diabetes and hypoalbuminemia at the time of hospitalization; (c) intracranial infection diagnosed before CP.

Demographic characteristics such as age, sex, indication, complications after DC, duration of skull defect, surgery duration, and amount of bleeding during CP were recorded. Patients were divided into younger group (≤40 years old) and older group (>40 years old). According to time interval, early repair was defined as cranioplasty less than 3 months after DC while late repair group was defined as cranioplasty at least 3 months after DC.

For skull shape changes, compared with the surrounding normal skull, herniation was defined as that the average protrusion of repair part is more than 1.5 cm, and skull depression was defined as that the average reduction of repair part is more than 1.5 cm. For a history of ventriculoperitoneal shunt (VPS), we defined it as VPS performed before CP and after DC. As for VPS after CP, VPS and cranioplasty performed on the same day were classified as concurrent surgery; on the contrary, VPS underwent a period after CP was defined as the staged surgery. As for hydrocephalus, we only recorded newly onset postoperative hydrocephalus, excluding those with hydrocephalus before the CP operation. Imaging data were recorded along with other perioperative parameters by two professional neurosurgeons. Postoperative complications were defined as complications after cranioplasty. Postoperative complications were recorded by comparing pre- and postoperative medical records and CT scan image including pneumocephalus, hydrocephalus, subdural and epidural effusion, subdural and epidural hematoma, seizures, intracranial and superficial infection, and implant failure included removal due to incision dehiscence or implant exposure and reoperation after severe complications. Similarly, it was defined as postoperative overall complications, as long as any of the above postoperative complications were contained.

Post-discharge complications were defined as complications after discharge. We followed up *via* telephone survey with a mean duration of 33.4 months (from 7.4 to 60.3 months) and collected information on post-discharge complications, including intracranial bleeding, epilepsy, subcutaneous effusion, shape change (sunken or herniated), and implant failure. Similarly, it was defined as post-discharge overall complications, as long as any of the above post-discharge complications were contained. We have a video call with the family members who are responsible for taking care of the patients to view the current physical condition and skull appearance of the patients in real time and back up all media materials provided by the family members after the patient's discharge, including photos, videos, imaging materials, etc. The degree of satisfaction after cranioplasty was also recorded based on the following score: (1) Poor, (2) Acceptable, (3) Excellent, according to complication, cosmesis, and patients' attitude of evaluation. For the results reported by the patients, patients gave an oral consent before participating in the survey. In the multiple regression analysis of the implant failure, intracranial infection after CP and the wound infection collected during follow-up were included. Among them, intracranial infection was the manifestation of brain inflammation and exudation in hospitalization records and imaging data. Superficial infection referred to the occurrence of suppuration in the wound photos provided by patients during follow-up, which might cause poor incision healing.

### Statistical analysis

#### Overall comparison by materials (PEEK vs. titanium mesh)

Baseline demographics, clinical variables, and complications after CP in both groups were compared using the χ2 test or Fisher exact test for categorical variables and Student's *t*-test for continuous variables.

#### Risk factors analysis in subgroups by complications

Univariate and multivariate logistic regression analyses were performed to explore potential risk factors in the overall complication group and each subgroup. All statistical analyses were performed with SPSS 26.0. Continuous variables are presented as mean ± SD, and categorical variables as percentages. Two-tailed *p*-values <0.05 were considered statistically significant.

## Results

### Patient demographics

This retrospective study included 211 patients who underwent CP, 105 using titanium mesh, and 106 using PEEK. The mean time interval of skull defect was 253.11 days, including 59 cases in the early period (less than 3 months) and 152 cases in the late period (over 3 months). More demographics and patients' characteristics are detailed in Table 1. Patients who underwent CP with PEEK were significantly younger than those in the titanium mesh group (*p* < 0.001), and there was a significant difference in surgery duration between the two groups (*p* = 0.041). It should be noted that in Table 2, the overall complication rate after DC in TI group (71.4 vs. 49.0%, *p* = 0.001) was higher than that in PEEK group, especially hydrocephalus (37.1 vs. 11.3%, *p* < 0.001) and secondary surgery (10.4 vs. 0.9%, *p* = 0.003), while the infection rate after DC was significantly lower in TI group than in PEEK group (9.4 vs. 17.8%, *p* = 0.004). No more complications after DC were statistically different between the groups.

**Table 1:** Patient demographics and characteristics between TI group and PEEK group.

	**TI *N =* 105 (%)**	**PEEK *N =* 106 (%)**	***p*-values**
Number of males	69 (65.7%)	80 (75.5%)	0.120
Mean age (y)	50.4 ± 15.3	32.8 ± 17.5	**<0.001**
Surgery duration (h)	2.9 ± 1.5	3.3 ± 1.4	**0.041**
Intraoperative blood loss (mL)	154.6 ± 107.0	207.2 ± 135.4	**0.002**
Indication of decompressive craniectomy			0.829
Trauma	50(47.6%)	57 (53.8%)	
Intracranial hemorrhage	46 (43.8%)	41 (38.7%)	
Cerebral infarction	4 (3.8%)	3 (2.8%)	
Intracranial tumor	5 (4.8%)	5 (4.7%)	
Site			0.367
Left	44 (41.9%)	43 (40.6%)	
Right	56 (53.3%)	51(48.1%)	
Bilateral	4 (3.8%)	9 (8.5%)	
Other	1 (1%)	3 (2.8%)	
Time of defect (d)	146.5 ± 130.2	256.6 ± 655.3	0.092

Bold represents the *p* values with statistical difference (*p* < 0.05).

**Table 2:** Complication after decompressive craniectomy between TI group and PEEK group.

	**TI *N =* 105 (%)**	**PEEK *N =* 106 (%)**	***p*-values**
Overall complication after DC^*^	75 (71.4%)	52 (49.0%)	**0.001**
Hydrocephalus	39 (37.1%)	12 (11.3%)	**<0.001**
Infection			0.639
Wound infection	3 (2.8%)	3 (2.8%)	
Intracranial infection	7 (6.6%)	4 (3.7%)	
Hemorrhage			0.075
Substantial bleeding	10 (9.5%)	3 (2.8%)	
Epidural hematoma	10 (9.5%)	16 (15.0%)	
Epilepsy	6 (5.7%)	2 (1.8%)	0.146
Effusion			0.282
Epidural effusion	1 (0.9%)	1 (0.9%)	
Subcutaneous effusion	2 (1.9%)	4 (3.7%)	
Subdural effusion	4 (3.8%)	10 (9.4%)	
Pneumocephalus	6 (5.7%)	2 (1.8%)	
Subdural hematoma	1 (0.9%)	0	
Implant failure	11 (10.4%)	1 (0.9%)	**0.003**
Skull shape change			0.496
Sinking	34 (32.3%)	27 (25.4%)	
Herniation	33 (31.4%)	34 (32.0%)	

* DC, decompressive craniectomy. Bold represents the *p* values with statistical difference (*p* < 0.05).

### Postoperative and post-discharge complications

#### Postoperative complications

The overall complication rates after titanium mesh and PEEK CP in our study were 51.4 and 38.7% (*p* = 0.063). The occurrence incidence of postoperative complications including pneumocephalus, hydrocephalus, intracranial infection, subdural hematoma, epidural hematoma, implant failure, epilepsy, subdural effusion, and epidural effusion are summarized in Table 3. The most common postoperative complication of CP was pneumocephalus (19.9% in all patients). Table 3 shows that the rate of pneumocephalus (33.3 vs. 6.6%, *p* < 0.001) and epidural effusion (18.0 vs. 6.6%, *p* = 0.011) in the titanium mesh group was significantly higher than those in the PEEK group. No other postoperative complications were statistically different between the groups.

**Table 3:** Postoperative complication between TI group and PEEK group.

	**TI *N =* 105 (%)**	**PEEK *N =* 106 (%)**	***p*-values**
**Postoperative overall complication**	54 (51.4%)	41 (38.7%)	0.063
Pneumocephalus	35 (33.3%)	7 (6.6%)	**<0.001**
Subcutaneous effusion	30 (28.6%)	21 (19.8%)	0.137
Hydrocephalus	6 (5.7%)	7 (6.6%)	0.788
Intracranial infection	5 (4.8%)	8 (7.5%)	0.400
Subdural hematoma	6 (5.7%)	11 (10.4%)	0.213
Epidural hematoma	6 (5.7%)	9 (8.5%)	0.433
Epilepsy	1 (1.0%)	4 (3.7%)	0.178
Implant failure	3 (2.8%)	4 (3.7%)	0.710
Subdural effusion	14 (13.3%)	12 (11.3%)	0.657
Epidural effusion	19 (18.0%)	7 (6.6%)	**0.011**

Bold represents the *p* values with statistical difference (*p* < 0.05).

#### Post-discharge complications

We successfully followed up 86 of 105 patients in the titanium mesh group and 98 of 106 patients in the PEEK group with a total response rate of 87.2% (Table 4). Patients in PEEK group were less likely to occur subcutaneous effusion after discharge than those in TI group (2.0 vs. 10.5%, *p* = 0.013). No other post-discharge complications were significantly different between the groups. In terms of follow-up, success rate was statistically significant in titanium mesh group and PEEK group (95.3 vs. 99.0%, *p* = 0.023). Among the 184 patients who were followed up successfully, titanium mesh failed three times and PEEK failed four times when the implants were removed (*p* = 0.873). No significant difference in the rate of satisfaction was found in the survey of satisfaction after cranioplasty (*p* = 0.160).

**Table 4:** Post-discharge complication between TI group and PEEK group.

	**TI *N =* 86 (%)**	**PEEK *N =* 98 (%)**	***p*-values**
**Post-discharge overall complications**	31 (36.0%)	34 (34.7%)	0.703
Intracranial bleeding	1 (1.2%)	1 (1.0%)	0.905
Epilepsy	22 (25.6%)	16 (16.3%)	0.092
Subcutaneous effusion	9 (10.5%)	2 (2.0%)	**0.013**
Skull shape change			0.499
Sinking	19 (22.1%)	17 (17.3%)	
Herniation	4 (4.7%)	3 (3.1%)	
Wound healing			0.332
Normal	77 (89.5%)	94 (96.0%)	
Poor	5 (5.8%)	3 (3.1%)	
Implant failure	3 (3.5%)	4 (4.1%)	0.873
Follow-up			
Success	82 (95.3%)	97 (99.0%)	
Death	4 (4.7%)	1 (1.0%)	
Rate of satisfaction			0.160
Excellent	50 (58.1%)	63 (64.3%)	
Acceptable	29 (33.7%)	34 (34.7%)	
Poor	3 (3.5%)	0	

Bold represents the *p* values with statistical difference (*p* < 0.05).

### Risk factors of postoperative overall complications

In univariate analysis, time of defect (*p* = 0.049) and a history of staged VPS before CP (*p* = 0.008) were associated with postoperative overall complications. The results of the multivariate logistic regression analysis of postoperative overall complications are displayed in Table 5. Repair materials and hydrocephalus after DC were also included in multivariate analysis, even if this factor was not statistically significant in univariate analysis (*p* = 0.063, *p* = 0.193, respectively). According to Table 5, only a history of VPS before CP was statistically significant (*p* = 0.023) in the multivariate model. No other factors were significantly associated with postoperative overall complications in this multivariate logistic analysis.

**Table 5:** Multivariate logistics regression analysis on postoperative overall complication.

**Clinical varieties**	**N**	**OR (95% CI)**	***p-*values**
Time of defect>90 days			
Yes	152	0.565 (0.301, 1.060)	0.075
No	59	1	
A history of VPS before CP^*^			
Yes	20	3.838 (1.205, 12.222)	**0.023**
No	191	1	
Repair materials			
TI	105	1	
PEEK	106	0.778 (0.428, 1.417)	0.412
Hydrocephalus after DC^*^			
Yes	51	0.968 (0.462, 2.027)	0.931
No	160	1	

* CP, cranioplasty.

VPS, ventriculoperitoneal shunt.

DC, decompressive craniectomy. Bold represents the *p* values with statistical difference (*p* < 0.05).

### Risk factors of post-discharge overall complications

The results of the multivariate logistic regression analysis of post-discharge overall complications are displayed in Table 6. No factors were significantly associated with post-discharge overall complications in multivariate logistic analysis.

**Table 6:** Multivariate logistics regression analysis on post-discharge overall complications.

**Clinical varieties**	**N**	**OR (95%CI)**	***p-*values**
VPS after CP^*^			
Without VPS	157	1	
Staged surgery	41	0.552 (0.059, 5.172)	0.603
Concurrent surgery	13	3.599 (0.808, 16.042)	0.093
Epidural hematoma			
Yes	15	0.390 (0.095, 1.600)	0.191
No	196	1	
Subdural hematoma			
Yes	17	1.907 (0.632, 5.755)	0.252
No	194	1	
Epilepsy			
Yes	5	7.528 (0.707, 80.150)	0.094
No	206	1	

* CP, cranioplasty.

VPS, ventriculoperitoneal shunt.

### Risk factors of each complication

In the pneumocephalus subgroup, repairing with titanium mesh and flap depression at the defect site before CP was significant in both univariate [(OR = 7.071, 95% = CI 2.971–16.833, *p* < 0.001), (OR = 0.207, 95% CI = 0.081–0.530, *p* = 0.001)] and multivariate logistic regression analyses (*p* < 0.001, *p* = 0.024, respectively). The results of multivariate analysis model for this subgroup are detailed in Table 7.

**Table 7:** Multivariate logistics regression analysis on pneumocephalus.

**Clinical varieties**	***N***	**OR (95%CI)**	***p*-values**
Age>40			
Yes	113	1.152 (0.483, 2.749)	0.749
No	98	1	
Time of defect>90 days			
Yes	152	0.811 (0.357, 1.843)	0.617
No	59	1	
A history of VPS before CP^*^			
Yes	20	1.697 (0.503, 5.724)	0.394
No	191	1	
Skull shape change after DC^*^			
Normal	83	1	
Herniation	67	0.529 (0.187, 1.495)	0.230
Sinking	61	2.745 (1.142, 6.599)	**0.024**
Repair materials			
TI	105	1	
PEEK	106	0.143 (0.054, 0.382)	**<0.001**
Hydrocephalus after DC^*^			
Yes	51	0.976 (0.380, 2.505)	0.960
No	160	1	

* DC, cranioplasty.

VPS, ventriculoperitoneal shunt.

DC, decompressive craniectomy. Bold represents the *p* values with statistical difference (*p* < 0.05).

We further explored potential risk factors for implant failure after CP. Superficial infection and intracranial infection were statistically significant in both univariate [(OR = 55.333, 95% CI = 10.258–298.470, *p* < 0.001), (OR = 37.333, 95%CI = 9.583–145.440, *p* < 0.001)] and multivariate analysis models (*p* < 0.001, *p* = 0.001, respectively) (Table 8).

**Table 8:** Multivariate logistics regression analysis on implant failure after CP.

**Clinical varieties**	**N**	**OR (95%CI)**	***p*-values**
Time of defect>90 days			
Yes	152	6.463 (0.349,119.690)	0.210
No	59	1	
Repair materials			
TI	105	1	
PEEK	106	1.161 (0.177, 7.617)	0.876
Intracranial infection			
Yes	11	22.710 (3.460, 149.060)	**0.001**
No	200	1	
Superficial infection			
Yes	6	63.042 (7.327, 542.423)	**<0.001**
No	205	1	

^*^VPS, ventriculoperitoneal shunt. Bold represents the *p* values with statistical difference (*p* < 0.05).

## Discussions

### Overall postoperative and post-discharge complication rates after CP

Limited studies have compared the short-term and long-term outcomes of PEEK and titanium implants (9, 17, 23–26). Our retrospective study of 211 cases in our center is one of the studies comparing titanium mesh and PEEK for CP under such a large sample size and shows similar overall postoperative complication rates (51.4 and 38.7%, *p* = 0.063). At present, existing studies (9, 23, 27) comparing the effect of PEEK and titanium mesh in CP have limitations such as small number of cases, incomplete types of complications involved, and lack of long-term follow-up or exploration of risk factors. The overall data show that the complication rate of CP is between 15 and 36.5% (28). The complication rate of CP with PEEK is lower than that of titanium mesh, the shaping effect of PEEK is better, and the degree of satisfaction is higher (26, 29, 30). Zhang (23) conducted a multicenter retrospective study and found that the complications in the PEEK group were significantly lower than those in the titanium mesh group (17.3 vs. 31.8%), and Thien (9) reported complication rates of 25.0 and 27.8% for PEEK and titanium cranioplasty without any significance, suggesting that the comparison of postoperative effect of the two materials is still under debate. Limited studies have carried out statistical analysis and discussion on long-term complications after discharge, most of which investigated merely the satisfaction degree or limited types of complication (23, 26, 30). As is seen from our results, the post-discharge overall complications rate in PEEK and TI groups was 34.7 and 36.0% (*p* = 0.703) with no significance. The rate of “excellent” and “acceptable” evaluation is a little bit higher in PEEK group (99.0 vs. 96.3%), similar to Asaad's result (100 vs. 99%) (26).

### Risk factors of overall CP complications and optimal time interval before cranioplasty

According to our results, a history of ventriculoperitoneal shunt (VPS) before CP was an independent risk factor for early overall complications, increasing the early complication rate of CP. Hirschmann (31) reported a significant increase in the incidence of epidural/subdural effusion and epidural/subdural hematoma in patients who underwent VPS before surgery. As for the reason, patients with a previous VPS are more likely to have skin flap depression, increasing the difficulty of exposing the dural scar layer with repeated contusion and stretching of subdural contents, leading to the risk of subdural hemorrhage and disturbance of cerebrospinal fluid (CSF) circulation, which facilitates the formation of epidural and subcutaneous effusions (31). Therefore, adjusting the amount and speed of drainage seems important; a programmable valve to reduce the volume of CSF drainage was suggested, and the patient's upper body should be positioned as flat as possible after surgery, with temporary ligation and shunt also being considered. However, Schuss (32) and Heo (33) recommended that shunt operation and repair should be performed at different stages rather than concurrently in patients requiring VPS, which significantly reduces postoperative complications. Hirschmann (31) and Meyer (34) reported no significant difference in the incidence of complications between the two groups. A recent study comparing effect of concurrent vs. staged VPS and CP suggests no significant difference in infections, resorption, and implant failure, although implant failure and hospital-acquired infection were lower in concurrent VPS group. In our study, as shown in Supplementary Tables 1, 2, the incidence of postoperative overall complications and post-discharge overall complications was similar in staged surgery group and concurrent surgery group [(55.6 vs. 36.4%, *p* = 0.391), (20.0 vs. 62.5%, *p* = 0.135), respectively]. However, considering the reduction in the number of surgeries, Rosinski (35) recommended concurrent but not staged procedure in VPS and CP.

Although the optional time interval after DC to perform CP was not an independent risk factor for overall postoperative complications, according to multivariate logistic regression analysis (*p* = 0.075), the complication rate of early CP (≤3 months) was significantly higher (55.9 vs. 40.8%, *p* = 0.047), which is shown in Supplementary Table 3, suggesting that early CP after DC may increase overall postoperative complications, consistent with the findings of Goedemans (36). Notably, the incidence of post-discharge subcutaneous effusion was higher in early repair group than that in late repair group (12.2 vs. 3.7%, *p* = 0.028) shown in Supplementary Table 4. Previous evidence shows that early repair is more effective than late construction (37, 38), but there are also opposing views (39, 40). Systematic reviews by Xu (41) and Malcolm (42) showed comparable complication rates between early and late CP, suggesting that early cranioplasty might increase the risk of hydrocephalus while might decrease extra-axial fluid collection in the trauma population. In addition, Yao (43) reported that early construction can reduce the incidence of postoperative epilepsy, but Morton (44) found that extending the time interval after craniotomy can reduce the risk of postoperative infection. According to our results, which were shown in Supplementary Table 5, the rates of postoperative complications and post-discharge complications were similar in three groups. Because there is no strong and thick fibrous forming between the brain tissue in the early period, it is easy to induce blood and fluid leakage after CP, causing bone window sinking, affecting CSF hemodynamics, increasing the difficulty and complications of late repair surgery (45). Therefore, CP procedure during 3–6 months after DC is a better choice which can not only restore the brain tissue to a stable state, reduce the subdural space, but also avoid long-term bone window depression. More studies are needed to determine the indications for early CP, and individualized consideration of time interval before CP is warranted.

### Single postoperative complications and related risk factors

The incidence of postoperative pneumocephalus in the PEEK group was significantly lower than that in the titanium mesh group (*p* < 0.001). Implant materials and preoperative sunken skin were the only two independent risk factors of postoperative pneumocephalus. Subdural gas, a normal finding after neurosurgery, is thought to be the most common form of postoperative pneumocephalus (46). In most cases in our center, the gas was benign and absorbed in a few days without treatment, and no patients were found with pneumocephalus during the followed-up period. However, pneumocephalus may still lead to headache, poor postoperative brain tissue recruitment, infection, and even some serious conditions such as tension pneumocephalus. The volume of cerebrospinal fluid is reduced abnormally after the brain tissue is compressed, which is caused by sunken skull defect, leading to lower intracranial pressure than the atmosphere, which aggravates the depression of brain tissue (47), and thus, gas can easily enter through the broken dural. The intracranial pressure should be modified before CP to promote the shape recovery of brain tissue, and the dura should be carefully separated and tightly sutured during surgery to reduce the space under the flap (40). More researches are needed to gain a deeper understanding of the difference in pneumatosis rates between PEEK and titanium mesh. No more risk factors for epidural effusion were founded in our study.

Intracranial infection and superficial infection after CP were independent risk factors for implant failure after CP. There were seven cases of early implant failure in two groups, of which six (85.7%) were due to infection (four of superficial infection and two of deep infection). After discharge, seven patients underwent secondary surgery (five patients with superficial l suppurative infection). By comparing the effect of titanium mesh and PEEK repair, Thein (9) described intracranial infection and infection of incisional wounds as high-risk factors for secondary operation in hospital after failure of titanium mesh or PEEK surgery, consistent with our conclusion. Tsang (48) showed that VPS before and after surgery was an independent risk factor for postoperative infection, which was not observed in our study. In summary, perioperative prevention of infections is important to avoid implant failure. Infection after CP can be controlled after debridement and application of antibiotics, while Veldeman recommended improving scalp incision with a question mark, ending behind the ear to reduce the local infection rate after CP (49).

### Single post-discharge complications and related risk factors

We did not find any significant risk factor related to post-discharge complications, but some deserve attention and discussion, such as the wound dehiscence and the exposure of the implants. In our result, all implant exposure occur before discharge. Three of six wound infections cause the disruption of wound and exposure, and all of them underwent a second operation. After the operation, the incision was cleaned in time, and the red and swollen pus of the incision was treated in time. Further researches are needed to explore exposure rates of different materials and methods to avoid the exposure of implants. Sun argues that patients who have allergies to four or even more metal, the probability of titanium plate exposure will increase significantly (50). Another important complication was the appearance change including local sunk that occurs higher than herniation. Although no significant difference was found in appearance change between two groups, the rates of local depression were higher than that of herniation in both PEEK group (22.1 vs. 4.7%) and TI group (17.3 vs. 3.1%). Depression mostly occurred in the temporal region due to gradual atrophy after complete separation of the temporal muscle during surgery. Brandicourt (30) proposed fat-filling technology to overcome the depression caused by temporal muscle atrophy. A monopolar scalpel should be avoided when separating the temporal muscle during surgery to avoid the reduction of blood supply and atrophy caused by electric cauterization of the temporal muscle according to our center experience.

### How to make choice between PEEK and titanium mesh

Our study found that the average age of patients in the PEEK group was significantly lower than that in the titanium mesh group, which is an interesting phenomenon. Considering the need of life quality and higher aesthetic requirements of young patients, PEEK is a new type of repair material with better histocompatibility, which can compensate for the shortcomings of titanium mesh. Although it is expensive, it has gained wide popularity among young patients and even children. Most studies show higher satisfaction on PEEK cranioplasty (23, 26, 30), and multiple meta-analyses have confirmed lower revision rate in PEEK CP (14). But is PEEK exactly always a better choice? The results of our retrospective study showed no significant difference in either early or late complications between two materials. In addition, there is no significant difference in cosmetic change and satisfaction degree. Moreover, surgery time is longer and the amount of intraoperative blood loss of the PEEK group was significantly higher than that of the titanium mesh group; because during PEEK CP, the edge of the bone window and the temporal muscle need to be completely separated, while in titanium mesh CP, they only need to be partially separated in a less complicated procedure. In addition, PEEK has a small pore size, large space distance, thick bone plate, and limited patency of fluid drainage between the inner dura and the outer musculocutaneous flap (51), which does not significantly reduce the risk of early subcutaneous effusion. According to Zhang (23), the cost of PEEK procedure is extremely higher than that of titanium mesh procedure (24844.88 vs. 6438.31$). Therefore, the choice of repair materials for patients with skull defects remains controversial, and it is time to assess repeatedly the clinical and cost-effectiveness of two materials. The topic is gaining increasing attention, and new multicenter researches have been proposed and are being conducted in China (22). More multicenter comparative research data are needed to confirm the optimal choice of CP materials. Further, the advantages and disadvantages of the two materials should be fully explained to the patients' families before surgery.

### Strengths and limitations

Compared with most existing clinical studies, the sample size of this single-center retrospective study was larger with 211 cases. The types of complications were more detailed, and more risk factors were taken into consideration to better reflect the real situation and short-term and long-term effects of cranioplasty with TI and PEEK. However, this study also has limitations related to the loss of follow-up data and inadequate follow-up duration with mean time of only 33.4 months. Some demographic data existed significant difference such as age, surgery duration, and time interval between cranioplasty and DC. However, these data also show the true situation of the application of titanium and PEEK which are important parameters to identify the repair effect and indications to choose these two different materials in order to help patients make choice more comprehensively. In addition, data such as whether there were complications such as pneumocephalus and types of effusion disappeared were not included. Further, this was a single-center retrospective study with an inherent risk of bias. In the future, more multicenter clinical studies with a larger sample size are warranted to further explore the related risk factors of postoperative complications of PEEK and titanium mesh CP, and more detailed information of each complication should be studied so as to provide a more comprehensive reference for the selection of clinical CP materials.

## Conclusion

In conclusion, we presented detailed information on the complications and related risk factors associated with PEEK and titanium cranioplasty after DC. There were no differences in overall postoperative and post-discharge complication rates between the titanium mesh and PEEK. But among postoperative complications, the incidence of pneumocephalus during hospitalization (33.3 vs. 6.6%, *p* < 0.001) and epidural effusion in the titanium mesh group were significantly higher than those in the PEEK group (18.0 vs. 6.6%, *p* = 0.011). In addition, among the post-discharge complications, patients in PEEK group were less likely to occur subcutaneous effusion after discharge than those in TI group (2.0 vs. 10.5%, *p* = 0.013). For multivariate analysis, a history of VPS before CP was an independent risk factor for postoperative overall complications, and infection was a risk factor for postoperative implant failure. Finally, depression skull defects and titanium mesh implants increased the incidence of postoperative pneumocephalus. Our results aim to promote a better understanding of PEEK and titanium cranioplasty and help both clinicians and patients make better choices in implant materials.

## Data availability statement

The original contributions presented in the study are included in the article/Supplementary material, further inquiries can be directed to the corresponding author.

## Ethics statement

The studies involving human participants were reviewed and approved by Medical Ethics Committee of Zhujiang Hospital of Southern Medical University. Written informed consent from the participants or the participants' legal guardian/next of kin was not required to participate in this study in accordance with the National Legislation and the Institutional requirements.

## Author contributions

SY, QZ, and YM contributed equally to drafting the original manuscript. HY, YL, and MZ completed the statistical analysis and critically revised the manuscript. RZ was responsible for project design and research cost management. All authors contributed to the article and approved the submitted version.

## Funding

This work was supported by grants from the National Natural Science Foundation of China (No.81701200) to RZ.

## Conflict of interest

The authors declare that the research was conducted in the absence of any commercial or financial relationships that could be construed as a potential conflict of interest.

## Publisher's note

All claims expressed in this article are solely those of the authors and do not necessarily represent those of their affiliated organizations, or those of the publisher, the editors and the reviewers. Any product that may be evaluated in this article, or claim that may be made by its manufacturer, is not guaranteed or endorsed by the publisher.
