# Supplementary material for: Outcome and risk factors of complications after cranioplasty with polyetheretherketone and titanium mesh: A single-center retrospective study
Source: Front Neurol. 2022 Sep 21;13:926436. doi: 10.3389/fneur.2022.926436 (PMC9533107; doi:10.3389/fneur.2022.926436)
Supplement: Supplementary file 1 [file Data_Sheet_1.pdf]

Supplementary Table 1. Postoperative complication between concurrent VPS group and staged VPS group

	Concurrent VPS* N=11（%）	Staged VPS* N=9（%）	P Values
**Postoperative overall complication**	4（36. 4%）	5（55.6%）	0.391
Pneumocephalus	1（9.1%）	2（22.2%）	0.413
Hydrocephalus	0	2（22.2%）	0.099
Intracranial infection	2（18.2%）	0	0.178
Epidural hematoma	1（9.1%）	1（11.1%）	0.881
Subdural effusion	3（27.3%）	0	0.089
Epidural effusion	0	2（22.2%）	0.099

*VPS: ventriculoperitoneal shunt

Supplementary Table 2. Post-discharge complication between concurrent VPS group and staged VPS group

	Concurrent VPS* N=8（%）	Staged VPS* N=5（%）	P Values
**Post-discharge overall complications**	5（62.5%）	1（20.0%）	0.135
**Epilepsy**	2（25.0%）	1（20.0%）	0.835
**Subcutaneous effusion**	2（25.0%）	0	0.224
**Skull shape change**			0.252
Sinking	1（12.5%）	2（40.0%）	
Herniation	0	0	
**Wound healing**			0.411
Normal	7（87.5%）	5（100.0%）	
Poor	1（12.5%）	0	
**Rate of satisfaction**			0.279
Excellent	4（50.0%）	4（80.0%）	
Acceptable	4（50.0%）	1（20.0%）	
Poor	0	0	

*VPS: ventriculoperitoneal shunt

Supplementary Table3. Postoperative complication between early repair group and late repair group

	Early repair N=59（%）	Late repair N=152（%）	P Values
**Postoperative overall complication**	33（55.9%）	62（40.8%）	**0.047**
Pneumocephalus	14（23.7%）	28（18.4%）	0.386
Hydrocephalus	6（10.1%）	7（4.6%）	0.131
Intracranial infection	4（6.7%）	9（5.9%）	0.816
Subdural hematoma	5（8.4%）	12（7.8%）	0.890
Epidural hematoma	4（6.7%）	11（7.2%）	0.908
Epilepsy	1（1.6%）	4（2.6%）	0.688
Implant failure	2（3.3%）	5（3.2%）	0.971
Subdural effusion	8（13.5%）	18（11.8%）	0.733
Epidural effusion	5（8.4%）	21（13.8%）	0.289

Supplementary Table 4. Post-discharge complication between early repair group and late repair group

	Early repair N=49（%）	Late repair N=135（%）	P Values
**Post-discharge overall complication**	19（38.8%）	46（34.1%）	0.495
**Intracranial bleeding**	1（2.0%）	1（0.7%）	0.443
**Epilepsy**	12（24.5%）	26（19.3%）	0.401
**Subcutaneous effusion**	6（12.2%）	5（3.7%）	**0.028**
**Skull shape change**			0.600
Sinking	8（16.3%）	28（20.7%）	
Herniation	1（2.0%）	6（4.4%）	
**Wound healing**			0.460
Normal	44（89.8%）	127（94.1%）	
Poor	3（6.1%）	5（3.7%）	
**Implant failure**	2（4.1%）	5（3.7%）	0.887
**Follow up**			0.423
Success	47（95.9%）	132（97.8%）	
Death	2（4.1%）	3（2.2%）	
**Rate of satisfaction**			0.532
Excellent	29（59.2%）	84（62.2%）	
Acceptable	18（36.7%）	45（33.3%）	
Poor	0	3（2.2%）	

Supplementary Table 5. Postoperative complications among groups with different interval between DC* and CP*

	less than 3 months N=59（%）	3-6 months N=92（%）	More than 6 months N=60（%）	F Values	P Values
**Postoperative overall complications**	33（55.9%)	39（42.4%)	23(38.3%)	2.101	0.124
**Postoperative implant failure**	2(3.3%）	4(4.3%)	1(1.6%)	0.814	0.368
**Post-discharge overall complications**	19(40.4%)	20(25.6%)	16(29.6%)	1.089	0.299
**Post-discharge implant failure**	2(4.3%）	1(1.3%)	4(7.4%)	3.318	0.071

*CP：cranioplasty
DC：decompressive craniectomy
